# No change in health-related quality of life for at-risk U.S. women and men starting HIV pre-exposure prophylaxis (PrEP): Findings from HPTN 069/ACTG A5305

**DOI:** 10.1371/journal.pone.0206577

**Published:** 2018-12-26

**Authors:** Shashi N. Kapadia, Chunyuan Wu, Kenneth H. Mayer, Timothy J. Wilkin, K. Rivet Amico, Raphael J. Landovitz, Adriana Andrade, Ying Q. Chen, Wairimu Chege, Marybeth McCauley, Roy M. Gulick, Bruce R. Schackman

**Affiliations:** 1 Division of Infectious Diseases, Weill Cornell Medicine, New York, New York, United States of America; 2 Department of Healthcare Policy and Research, Weill Cornell Medicine, New York, New York, United States of America; 3 Statistical Center for HIV/AIDS Research and Prevention, Fred Hutchinson Cancer Research Center, Seattle, Washington, United States of America; 4 Fenway Health, Department of Medicine, Beth Israel Deaconess Medical Center / Harvard Medical School, Boston, Massachusetts, United States of America; 5 Department of Health Behavior and Health Education, University of Michigan School of Public Health, Ann Arbor, Michigan, United States of America; 6 UCLA Center for Clinical AIDS Research & Education, University of California Los Angeles, Los Angeles, California, United States of America; 7 Department of Medicine, Johns Hopkins University School of Medicine, Baltimore, Maryland, United States of America; 8 Division of AIDS, National Institute of Allergy and Infectious Diseases, National Institutes of Health, Rockville, Maryland, United States of America; 9 FHI 360, Washington, DC, United States of America; Janssen Research and Development, UNITED STATES

## Abstract

**Introduction:**

Tenofovir (TDF)-containing PrEP is effective for HIV prevention, but its effect on health-related quality of life (QOL) is unknown. Using data from HPTN 069/ACTG A5305, a randomized study of potential PrEP regimens comparing maraviroc alone, or together with TDF or emtricitabine (FTC), to TDF + FTC (control), we evaluated the impact of these regimens on QOL in at-risk HIV-uninfected U.S. women and men.

**Methods:**

QOL was measured at baseline (before starting medications) and every 8 weeks through week 48 using the EQ-5D-3L. Responses were converted to a scale from 0.0 (death) to 1.0 (perfect health), using published valuation weights. Mean scores were compared between groups at each time point using nonparametric testing. Multivariable linear regression was used to adjust for potential confounders.

**Results:**

We analyzed 186 women (median age 35 years, 65% black, 17% Hispanic) and 405 men (median age 30 years, 28% black, 22% Hispanic), including 9 transgender participants analyzed based on sex-at-birth. Mean baseline QOL was 0.91 for women and 0.95 for men. There were minimal changes in mean QOL over time for any regimen (women: p = 0.29; men: p = 0.14). There were no significant differences between participants who continued the regimen compared to participants who discontinued early (women: p = 0.61; men: p = 0.1). Mean QOL did not differ significantly by regimen at any time point, both unadjusted and after adjustment for age, race/ethnicity, adherence, and use of alcohol, marijuana, opiates, and other substances.

**Conclusions:**

QOL in at-risk individuals starting candidate PrEP regimens in a clinical trial is similar to the general population and maintained over time. This finding did not vary among regimens or when adjusted for demographics, adherence, and substance use. Our findings are the first to show that starting a candidate PrEP regimen in at-risk HIV-uninfected U.S. women and men was not associated with significant changes in QOL.

**Trial registration:**

Clinicaltrials.gov NCT01505114.

## Introduction

Tenofovir disoproxil fumarate (TDF)-containing antiretroviral (ARV) regimens are safe and effective as pre-exposure prophylaxis (PrEP) to prevent HIV infection in at-risk individuals [1–3] and are recommended in current guidelines [4–7]. However, daily ARV for PrEP use may have an effect on health-related quality of life (QOL).

While there is a large body of literature on QOL in people living with HIV taking ARVs, the potential QOL effect of ARV administration for prevention on HIV-uninfected individuals is not known. Results from qualitative and survey studies suggest that medication toxicity, the burden of daily pill-taking, and stigma associated with ARV use may decrease QOL, which has contributed to patient and provider concerns about PrEP [8, 9]. Conversely, receipt of PrEP may reduce the anxiety and fear associated with HIV acquisition, and in that way positively affect QOL [9, 10]. No clinical studies of PrEP have reported the QOL impact of uninfected individuals taking ARV regimens for HIV prevention.

Using data from HPTN 069/ACTG A5305, a randomized phase 2 safety trial comparing 3 candidate PrEP regimens to a control regimen with TDF and emtricitabine (FTC), we evaluated the impact of ARV administration on QOL in U.S. individuals at-risk for HIV acquisition, assessed for differences between candidate PrEP regimens, and investigated factors associated with QOL in this population.

## Methods

### Design

HPTN 069/ACTG A5305 was a phase 2 randomized, double-blinded controlled safety and tolerability trial comparing maraviroc (MVC), MVC with FTC, MVC with TDF, and TDF with FTC (control) for HIV prevention.[11, 12] The study was approved by the institutional review boards at each participating site, and all participants provided written informed consent. The names of the individual institutional review boards that approved the study are listed in the supplementary information files (S1 Table).

### Participant selection

Study participants were enrolled between July 2012 and December 2014, with follow-up concluding in November 2015. The study population included HIV-uninfected women, men who have sex with men (MSM), and transgender individuals in the United States who were at risk for HIV acquisition based on self-reported condomless intercourse in the past 90 days with a male partner who was either HIV positive or of unknown serostatus [11, 12].

### Measurement of QOL

QOL was measured with the EQ-5D-3L, a validated instrument for QOL [13]. Survey responses were converted to health utilities, a preference-weighted measure of health status, on a scale from 0.0 (death) to 1.0 (perfect health) by using U.S. population valuation weights [14]. We also evaluated the EQ-5D Visual Analogue Scale (VAS), respondents’ self-rated perception of their health status on a 0 to 100 scale, with 0 as the worst health the respondent can imagine, and 100 as the best health the respondent can imagine. QOL was assessed at baseline (prior to initiating PrEP study drugs) and every 8 weeks through week 48. Participants who discontinued study drugs but remained in study also completed QOL assessments.

### Statistical analysis

The clinical trials recruited, randomized, and analyzed men and women in separate groups. In this manuscript, to be consistent with those trials, we analyze and report QOL results separately based on sex-at-birth.[11, 12] Mean scores were compared among regimens using nonparametric testing. Multivariable linear regression was used to adjust for demographics, substance use, and participants’ self-reported ability to take the regimen as prescribed. We used a linear regression model based on previous literature suggesting that these models perform similarly to other modeling approaches.[15] Analyses were conducted in SAS Version 9.4.

## Results

A total of 594 participants (188 women and 406 men) were enrolled in the study. Two women and one man were excluded from the QOL analysis due to failure to complete the baseline QOL assessment. We analyzed data from 186 women (median age 35 years, age range 18–61 years, 65% black, 17% Hispanic) and 405 men (median age 30 years, age range 18–70 years, 28% black, 22% Hispanic). Seven transgender women and two transgender men were grouped according to sex-at-birth. Baseline characteristics of study participants are shown in Table 1, stratified by treatment group. Among the women, 160 (86%) completed follow-up and 115 (62%) remained on their study regimens for all 48 weeks. Among the men, 343 (84%) completed study follow up, and 281 (69%) remained on study regimens. Table 2 shows the number of participants that discontinued study medications or missed the QOL assessment at each time point.

**Table 1:** Baseline characteristics stratified by sex and randomized regimen.

	Women (n = 186)					Men (n = 405)				
	MVC (n = 45)	MVC+TDF (n = 49)	MVC+FTC (n = 45)	TDF+FTC (n = 47)	Total (n = 186)	MVC (n = 101)	MVC+TDF (n = 99)	MVC+FTC (n = 105)	TDF+FTC (n = 100)	Total (n = 405)
**Median age (range)**	39 (18–61)	35 (22–60)	35 (19–57)	35 (18–60)	35 (18–61)	30 (18–65)	30 (18–70)	29 (18–62)	31 (18–60)	30 (18–70)
**Black Race: (%)**	29 (64.4)	32 (65.3)	31 (68.9)	29 (61.7)	121 (65.1)	31 (30.7)	30 (30.3)	33 (31.4)	21 (21.0)	115 (28.4)
**Alcohol Use: (%)**										
Daily	3 (6.7)	2 (4.2)	3 (6.8)	1 (2.1)	9 (4.9)	11 (10.9)	13 (13.1)	18 (17.1)	26 (26.0)	68 (16.8)
Yes, but not daily	28 (62.2)	33 (68.8)	30 (68.2)	35 (74.5)	126 (68.5)	78 (77.2)	77 (77.8)	72 (68.6)	59 (59.0)	286 (70.6)
No	14 (31.1)	13 (27.1)	11 (25.0)	11 (23.4)	49 (26.6)	12 (11.9)	9 (9.1)	15 (14.3)	15 (15.0)	51 (12.6)
**Marijuana Use: (%)**										
Daily	3 (6.8)	3 (6.1)	7 (15.9)	4 (8.9)	17 (9.3)	21 (20.8)	14 (14.1)	7 (6.7)	13 (13.0)	55 (13.6)
Yes, but not daily	6 (13.6)	17 (34.7)	11 (25.0)	12 (26.7)	46 (25.3)	30 (29.7)	33 (33.3)	50 (47.6)	27 (27.0)	140 (34.6)
No	35 (79.5)	29 (59.2)	26 (59.1)	29 (64.4)	119 (65.4)	50 (49.5)	52 (52.5)	48 (45.7)	60 (60.0)	210 (51.9)
**Opiate Use: (%)**										
Ever	4 (8.9)	2 (4.1)	3 (7.0)	6 (12.8)	15 (8.2)	11 (10.9)	4 (4.0)	10 (9.5)	10 (10.0)	35 (8.6)
Never	41 (91.1)	47 (95.9)	40 (93.0)	41 (87.2)	169 (91.8)	90 (89.1)	95 (96.0)	95 (90.5)	90 (90.0)	370 (91.4)
**Other substance use: (%)**										
Ever	9 (20.5)	8 (16.3)	12 (27.9)	13 (27.7)	42 (23.0)	57 (56.4)	45 (45.5)	52 (49.5)	57 (57.0)	211 (52.1)
Never	35 (79.5)	41 (83.7)	31 (72.1)	34 (72.3)	141 (77.0)	44 (43.6)	54 (54.5)	53 (50.5)	43 (43.0)	194 (47.9)

MVC = maraviroc, TDF = tenofovir disaproxil fumarate, FTC = emtricitabine

**Table 2:** Number of participants that discontinued regimens or missed quality of life assessment.

**Women**			
	**On Medications, Provided QOL**	**Off Medications, Provided QOL**	**Off Medications, Did not provide QOL**
**Week 0**	186	0	0
**Week 8**	169	10	7
**Week 16**	152	13	21
**Week 24**	144	19	23
**Week 32**	132	23	31
**Week 40**	130	28	28
**Week 48**	130	30	26
**Men**			
	**On Medications, Provided QOL**	**Off Medications, Provided QOL**	**Off Medications, Did not provide QOL**
**Week 0**	405	0	0
**Week 8**	379	5	20
**Week 16**	366	11	28
**Week 24**	350	17	38
**Week 32**	336	17	52
**Week 40**	326	23	56
**Week 48**	319	24	62

**Fig 1:**
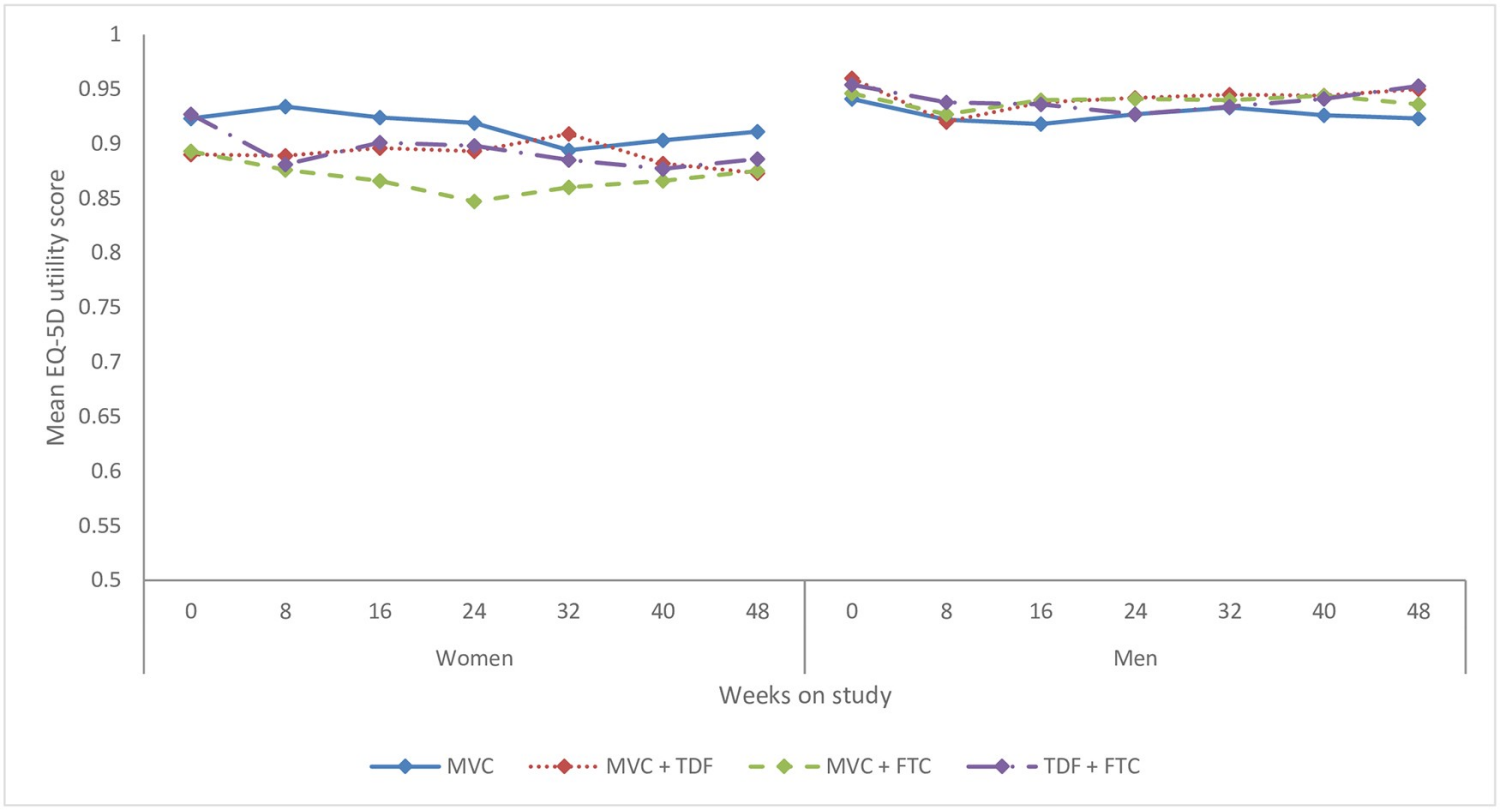
Mean EQ-5D utility (Quality of life) score by PrEP study regimen over time. Legend: Mean utility measured by EQ-5D, converted to health utility using U.S. valuation weights, where 0 is equivalent to death and 1 to perfect health. MVC = maraviroc, TDF = tenofovir disoproxil fumarate, FTC = emtricitabine.

There was no significant change in QOL score between the baseline assessment and any time during or at the end of the study. The mean QOL score for women was 0.91 (95%CI: 0.89–0.93) at pre-PrEP baseline and 0.89 (95%CI: 0.86–0.91) at week 48 (p = 0.29). The mean score for men was 0.95 (95%CI: 0.94–0.96) at pre-PrEP baseline and 0.94 (95%CI: 0.93–0.95) at week 48 (p = 0.14). Pre-PrEP baseline QOL scores were similar across the four ARV PrEP regimens, and there were minimal changes over time for any regimen. (Fig 1).

There was no significant difference in QOL at the end of the study among participants who stayed on the study regimen, participants who discontinued and restarted the regimen during the trial, and participants who discontinued the regimen early but continued study follow-up (women: p = 0.61; men: p = 0.1) (Table 3).

**Table 3:** Mean EQ-5D utility (Quality of life) score at week 48 by continuation of study regimen.

	Women			Men		
	N	Mean Utility	95% CI	N	Mean Utility	95% CI
**Stayed on regimen**	113	0.88	0.85–0.91	281	0.95	0.93–0.96
**Discontinued and restarted regimen**	15	0.90	0.82–0.98	38	0.95	0.91–0.98
**Off regimen**	30	0.91	0.86–0.96	24	0.87	0.78–0.95

Mean Utility measured by EQ-5D, converted to health utility using U.S. valuation weights. Treatment status measured at week 48.

In multivariate analyses, there was no difference in mean QOL among regimens at any time point when adjusted for age, race/ethnicity, alcohol use, marijuana use, opiate use, other substance use, or the most recent self-reported adherence assessment. Higher QOL was associated with self-reported greater ability to take the regimen as prescribed in both women and men at most time points. At week 48, women with a high ability to take the regimen had a higher QOL than those without high ability (β = 0.08, 95% CI [0.01, 0.15], p = 0.04). For men at week 48, there was a higher QOL associated with high ability (β = 0.03, 95% CI [-0.01, 0.07], p = 0.1). Each year of increased age was associated with lower baseline QOL in women (β = -0.002, 95% CI [-0.004, -0.001], p = 0.007), but not in men.

**Fig 2:**
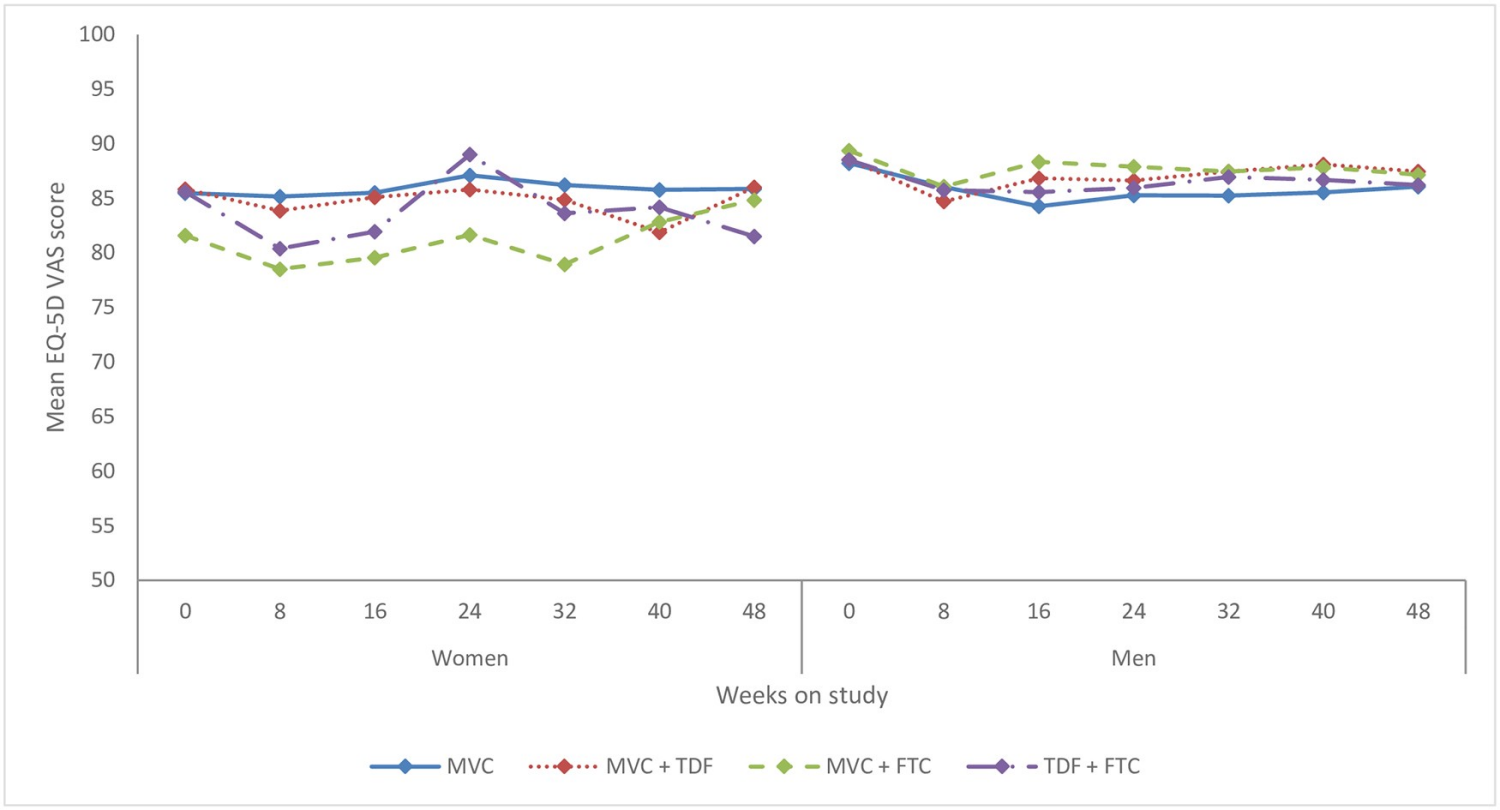
Mean EQ-5D visual analogue scale (VAS) score by PrEP study regimen over time. Mean utility measured by EQ-5D VAS, where 0 is the worst health the respondent can imagine, and 100 is the best. MVC = maraviroc, TDF = tenofovir disoproxil fumarate, FTC = emtricitabine.

Results using the EQ-5D Visual Analog Scale (VAS) were similar to those using the EQ-5D utility scores. Mean scores for women changed from 84.7 (95%CI: 82.5–86.9) at pre-PrEP baseline to 84.5 (95%CI: 82.2–86.9) at week 48. For men, mean VAS score was 88.7 (95%CI: 87.8–89.5) at baseline and 86.7 (95%CI: 85.5–87.9) at the end of study. Fig 2 shows changes in mean EQ-5D VAS over time for each treatment group and sex.

## Discussion

Our findings show that QOL in at-risk individuals prior to starting candidate HIV PrEP regimens in a clinical trial was similar to published values for the U.S. population of comparable age [16]. QOL was maintained at a high level during the period of candidate PrEP administration for all regimens. Higher baseline QOL was associated with younger age in women. During the study, higher QOL was associated with a high self-reported ability to take medication as prescribed. While the clinical significance of the differences in QOL as a result of these factors is uncertain, previous studies have indicated that the minimally important difference for EQ-5D scores using US valuation weights is 0.04.[17]

The stability of QOL over time was maintained for each of the maraviroc-containing regimens and also for TDF/FTC, which is currently the US Food and Drug Administration-approved PrEP standard of care. Our findings show that QOL as a global construct is not impacted by PrEP administration, a message important for both clinicians and at-risk individuals. Also, this finding may have implications for cost-effectiveness evaluation of PrEP regimens that include health utility as an outcome. However, specific domains of well-being, such as sexual well-being and anxiety related to HIV acquisition, which may be positively impacted by PrEP administration, were not specifically measured in the current analysis.

This study had several limitations. The study regimen comprised 3 pills (vs. 1 pill for the U.S. FDA approved TDF/FTC HIV PrEP regimen); the efficacy of MVC-containing regimens for HIV-prevention is not known and these regimens are not approved for HIV PrEP. Results in our study population may not be fully generalizable to the population of individuals who take PrEP. The EQ-5D may not be sensitive to small differences in QOL and may have ceiling effects for individuals in good health. [15] Our modeling approach may not account for regression to the mean. Associations between QOL and adherence or demographic factors may not be causal, as unmeasured confounders may affect these relationships. Missing observations from participants who did not appear for study follow-up may be different from those that remained in follow-up. Per the design of HPTN 069/ACTG A5305, at-risk heterosexual men were not included in the study; and a low number of transgender participants precluded analysis of that subgroup.

## Conclusions

Our findings are the first to show that starting a candidate PrEP regimen in a randomized clinical trial of at-risk HIV-uninfected U.S. women and men was not associated with significant changes in QOL.

## Supporting information

S1 Table List of 11 institutional review boards which approved the study. (DOCX)

S1 Dataset ptn069_qol.zip. (ZIP)
