# Supplementary material for: No change in health-related quality of life for at-risk U.S. women and men starting HIV pre-exposure prophylaxis (PrEP): Findings from HPTN 069/ACTG A5305
Source: PLoS One. 2018 Dec 26;13(12):e0206577. doi: 10.1371/journal.pone.0206577 (PMC6306196; doi:10.1371/journal.pone.0206577)
Supplement: S1 Table — (DOCX) [file pone.0206577.s001.docx]

Title: No change in health-related quality of life for at-risk U.S. women and men starting HIV pre-exposure prophylaxis (PrEP): Findings from HPTN 069/ACTG A5305

Authors: Shashi N Kapadia, Chunyuan Wu, Kenneth H Mayer, Timothy J Wilkin, K. Rivet Amico, Raphael J Landovitz, Adriana Andrade, Ying Q Chen, Wairimu Chege, Marybeth McCauley, Roy M Gulick, Bruce R Schackman

Supplementary Information File 1:

**Ethics Statement:**

The study was approved by the institutional review boards at each participating site, and all participants provided written informed consent.

The 11 Institutional Review Boards that approved the study are as follows:

1. Fenway Institute, Boston MA 02215

2. George Washington University, Washington DC 20037

3. San Francisco Department of Public Health HIV Research Section, San Francisco CA 94102

4. University of North Carolina, Chapel Hill NC 27599

5. Case Western Reserve University, Cleveland OH 44106

6. Weill Cornell Medicine, New York NY 10011

7. Johns Hopkins University, Baltimore MD

8. University of Pennsylvania, Philadelphia PA 19104

9. University of Pittsburgh, Pittsburgh PA 15213

10. University of Puerto Rico, San Juan PR

11. University of California Los Angeles, Los Angeles CA 90035
