# Supplementary material for: No change in health-related quality of life for at-risk U.S. women and men starting HIV pre-exposure prophylaxis (PrEP): Findings from HPTN 069/ACTG A5305
Source: PLoS One. 2018 Dec 26;13(12):e0206577. doi: 10.1371/journal.pone.0206577 (PMC6306196; doi:10.1371/journal.pone.0206577)
Supplement: S1 Dataset — (ZIP) [file pone.0206577.s002.zip › ptn069_qol/data_dictionary_analysis_dataset.docx]

| **Variable Names** | **Format** | **Description** |  |
| --- | --- | --- | --- |
| Uid |  | Participant id | |
| Visit | 201=Baseline  501=Week 8  601=week 16  701=week 24  801=week 32  901=week 40  1001=week 48 | Visit | |
| Race_black | 1=Black  0=Non-Black | Race | |
| Arm_r | 1=MVC only  2=MVC + FTC  3=MVC + TDF  4=TDF + FTC | Arm | |
| Demsex | 1=Male  2=Female | Sex at Birth | |
| Age |  | Age |  |
| ACADHEREx | 1=Very Poor/Poor/Fair  2=Good/Very Good/Excellent  .= Missing/Declined to Answer | Please rate your ability to take your study medications every day in the past month. |  |
| ALCx | 1=Never  2=Yes, but not daily  3=Daily | Alcohol Use |  |
| MRJx | 1=Never  2=Yes, but not daily  3=daily | Marijuana Use | |
| OPLx | 0=Never  1=ever | Opiate Use |  |
| OSUB | 0=Never  1=ever | Other Substance Use |  |
| EQ_index2005 |  | EQ-5D Utility (Quality of life) score |  |
| AC_HLTH |  | visual analogue scale (VAS) score |  |
