# Supplementary material for: No change in health-related quality of life for at-risk U.S. women and men starting HIV pre-exposure prophylaxis (PrEP): Findings from HPTN 069/ACTG A5305
Source: PLoS One. 2018 Dec 26;13(12):e0206577. doi: 10.1371/journal.pone.0206577 (PMC6306196; doi:10.1371/journal.pone.0206577)
Supplement: S1 Dataset — (ZIP) [file pone.0206577.s002.zip › ptn069_qol/data_dictionary_discontinuation.docx]

| **Variable Names** | **Format** | **Description** |  |
| --- | --- | --- | --- |
| Uid |  | Participant id | |
| Visit | 201=Baseline  501=Week 8  601=week 16  701=week 24  801=week 32  901=week 40  1001=week 48 | Visit | |
| Arm_r | 1=MVC only  2=MVC + FTC  3=MVC + TDF  4=TDF + FTC | Arm | |
| Demsex | 1=Male  2=Female | Sex at Birth | |
| EQ_index2005 |  | EQ-5D Utility (Quality of life) score |  |
| rhdrsn | 1=study regimen related toxicity  2=abnormal lab value  3=clinical reasons determined by the investigator  4=Hepatitis B infection  5=One or more reactive HIV test results/possible acute HIV infection  6=reported use of prohibited concomitant medication  7=reported use of post-exposure prophylaxis  8=request by participant to terminate study regimen  9=participant is unwilling or unable to comply with required procedures  10=other  11=pregnancy | Why is the study regimen being held or discontinued? |  |
| rhdrsm | 1=yes  2=no (permanently discontinued)  3=no (hold continuing/permanently discontinued for another reason) | Was the participant instructed to resume study regimen use |  |
| On_trt | 1=yes, never discontinued  2=yes, discontinued and resumed  3=No | On and off treatment status |  |
